# 446. Prognostic Value of Absolute Lymphocyte Count for Disease Severity and Clinical Outcomes in Adult COVID-19 Inpatients

**DOI:** 10.1093/ofid/ofab466.645

**Published:** 2021-12-04

**Authors:** Jianli Niu, Candice Sareli, Maria Deane, Aharon E Sareli

**Affiliations:** Memorial Healthcare System, Hollywood, FL

## Abstract

**Background:**

Lymphopenia has been reported as a relatively frequent finding in patients with coronavirus disease 2019 (COVID-19). This study aimed to assess the use of absolute lymphocyte count (ALC) as a prognostic biomarker for disease severity and clinical outcomes.

**Methods:**

A cohort of adult patients with COVID-19 admitted to Memorial Healthcare System, Hollywood, Florida from March 7, 2020 to January 18, 2021 was retrospectively analyzed. An absolute lymphocyte count (ALC) < 1.1 × 10^9^/L was used as cutoff point to define lymphopenia. Correlations of ALC upon admission with age and serum levels of C-reactive protein, interleukin-6, lactate dehydrogenase, and creatinine were analyzed. Univariate and multivariate regression models were developed to assess the association of lymphopenia with the risk of ICU admission and clinical outcomes.

**Results:**

4,485 hospitalized patients were included in the final analyses. Median age was 61 (interquartile range, 47-73) years and 2,311 (51.5%) were men. Lymphopenia was more frequent in patients admitted to the ICU compared to those that were not admitted to the ICU, with an odds ratio of 2.14 (95% conﬁdence interval [CI], 1.78-2.56, p < .0001) (Figure 1). The actual value of the ALC was negatively correlated with age and serum levels of C-reactive protein, interleukin-6, lactate dehydrogenase, and creatinine (all p < 0.005). Patients with lymphopenia (n=2,409) compared to those without lymphopenia (n=2,076) had multivariable-adjusted odds ratios of 1.85 (95% CI, 1.53-2.24) for ICU admission, 2.08 (95% CI, 1.67-2.58) for intubation, 1.98 (95% CI, 1.31-3.00) for development of acute kidney failure, and 2.23 (95% CI, 1.79-2.79) for in-hospital mortality (Table 1). Analyses were adjusted for age, gender, race, hypertension, diabetes, chronic obstructive pulmonary disease, chronic kidney disease, coronary artery disease, malignancy, obesity, and smoking.

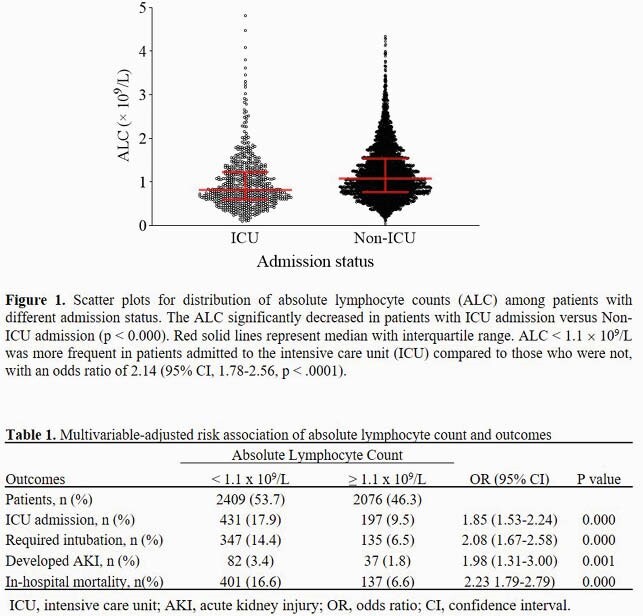

**Conclusion:**

Lymphopenia in adult COVID -19 hospitalized patients was associated with increased risk of disease severity (as evidenced by need for ICU admission) and poor clinical outcomes. Absolute lymphocyte count may help with prognostication in individuals hospitalized with COVID-19.

**Disclosures:**

**All Authors**: No reported disclosures

